# Legumes display common and host-specific responses to the rhizobial cellulase CelC2 during primary symbiotic infection

**DOI:** 10.1038/s41598-019-50337-3

**Published:** 2019-09-25

**Authors:** E. Menéndez, M. Robledo, J. I. Jiménez-Zurdo, E. Velázquez, R. Rivas, J. D. Murray, P. F. Mateos

**Affiliations:** 1 Departamento de Microbiología y Genética, Universidad de Salamanca, Salamanca, Spain; 2 Instituto Hispano-Luso de Investigaciones Agrarias (CIALE), Universidad de Salamanca, Salamanca, Spain; 3 Estación Experimental del Zaidín, CSIC, Granada, Spain; 4 Unidad asociada de I + D IRNASA-CSIC, Salamanca, Spain; 5 Department of Cell and Development Biology, John Innes Centre, Norwich, UK; 6 Present Address: Instituto de Ciências Agrárias e Ambientais Mediterrânicas (ICAAM), Universidade de Évora, Évora, Portugal; 7 Present Address: Instituto de Biomedicina y Biotecnología de Cantabria, Santander, Spain; 8 Present Address: Centre of Excellence for Plant and Microbial Science, Shangai, China

**Keywords:** Bacterial host response, Rhizobial symbiosis

## Abstract

Primary infection of legumes by rhizobia involves the controlled localized enzymatic breakdown of cell walls at root hair tips. Previous studies determined the role of rhizobial CelC2 cellulase in different steps of the symbiotic interaction *Rhizobium leguminosarum-Trifolium repens*. Recent findings also showed that CelC2 influences early signalling events in the *Ensifer meliloti-Medicago truncatula* interaction. Here, we have monitored the root hair phenotypes of two legume plants, *T. repens* and *M. sativa*, upon inoculation with strains of their cognate and non-cognate rhizobial species, *R. leguminosarum* bv trifolii and *E. meliloti*, (over)expressing the CelC2 coding gene, *celC*. Regardless of the host, CelC2 specifically elicited ‘hole-on-the-tip’ events (Hot phenotype) in the root hair apex, consistent with the role of this endoglucanase in eroding the noncrystalline cellulose found in polarly growing cell walls. Overproduction of CelC2 also increased root hair tip redirections (RaT phenotype) events in both cognate and non-cognate hosts. Interestingly, heterologous *celC* expression also induced non-canonical alterations in ROS (Reactive Oxygen Species) homeostasis at root hair tips of *Trifolium* and *Medicago*. These results suggest the concurrence of shared unspecific and host-related plant responses to CelC2 during early steps of symbiotic rhizobial infection. Our data thus identify CelC2 cellulase as an important determinant of events underlying early infection of the legume host by rhizobia.

## Introduction

The symbiotic relationship between a legume and its compatible rhizobia is a complex and highly regulated process involving early signalling steps for specific recognition between both partners. This infection process culminates with the bacterial-plant nutrient exchange that supports nitrogen fixation within the newly formed root-nodule organ^1–5^. Briefly, under low nitrogen conditions, legume roots exude flavonoids and, in return, rhizobia secrete Nod Factors (NFs), which are perceived by epidermal cells^1^. Bacteria attach to root hair tips, which curl to entrap bacteria within the so-called infection pockets^6,7^. Concomitantly, nodule formation is initiated in the root cortex by multiplication of meristematic cells. Rhizobia travel from the curled root hairs to the nodule primordia through a newly-formed tubular structure called the infection thread (IT), which is formed by invagination of the plant cell membrane^7^. Once in the nodule, the microorganisms are released in membrane-bound compartments inside the plant cells and commence their differentiation into nitrogen-fixing functional bacteroids.

Several studies have shown that rhizobia produce enzymes capable of degrading plant cell-wall polymers, which constitute the primary barrier for host infection^8–13^. In particular, *Rhizobium leguminosarum* bv trifolii ANU843 synthesize a ß-(1–4)-endoglucanase, called cellulase CelC2, that has been biochemically and functionally characterized. CelC2 is involved in the establishment of the symbiosis between this bacterium and its cognate host, white clover (*Trifolium repens*)^10^. Purified cellulase CelC2 degrades the isotropic apex of *T. repens* root hair tips *in vivo*, which is referred to as the HoT (Hole on the Tip) phenotype^9^. Interestingly, CelC2 erosion sites showed noncrystalline cellulose architecture, as they are located at the points of *de novo* synthesis of cell wall. Knockout mutants lacking the CelC2-encoding gene (*celC*), attach to root hairs but are unable to penetrate through the cell wall, aborting IT formation^10^. Furthermore, inoculation with a *celC*-overexpressing (CelC2^+^) derivative leads to aberrant symbiotic phenotypes in *T. repens*, i.e. extensive degradation of the IT origin and terminus loci^12^. Inoculation of *Medicago truncatula* with *Ensifer meliloti* (formerly *Sinorhizobium meliloti*) heterologously expressing CelC2 also altered signalling and nodulation^13^.

Host specificity is evident during the first stages in the establishment of an effective legume-rhizobia symbiotic interaction, i.e. a given rhizobial species typically recognizes and nodulates a narrow range of plant hosts. The key early determinants of specifity are thought to be flavonoids and Nod factors (NFs). The former are secreted by the host and trigger rhizobia production of NFs, which are in turn specifically recognized by the legume leading to IT formation and nodule organogenesis^1,14^. Other crucial core plant genetic determinants have been also related to NF-signalling pathway, i.e.: *LYK3, NFP, NSP1, NSP2, NIN, ERN1*^2,3,5,6^. Redox homeostasis signals, such as ROS and NO, are also involved in several steps through the rhizobia-legume interaction^15^. ROS accumulation plays important roles in the polar growth of root hairs and is necessary for infection initiation^7,16,17^. Moreover, other bacterial molecules have also been identified as determinants of symbiotic specificity, such as exopolysaccharides and specific secreted proteins^14,18–20^. Amongst them, the role of cell-wall hydrolytic and/or remodelling enzymes during symbiotic and pathogenic interactions has been extensively discussed^8–10,12,21–29^. However, the mechanisms underlying substrate specificity of bacterial cell-wall hydrolytic enzymes and their impact, if any, on host root infection remains unknown.

In the present study, we have monitored early responses of *Trifolium* and *Medicago* root hairs to inoculation with *R. leguminosarum* bv trifolii and *E. meliloti* wild-type and *celC*-expressing strains by microscopic and molecular techniques. These cross-inoculation experiments uncovered CelC2-dependent expression of specific and common host symbiotic traits.

## Experimental Procedures

### Bacterial strains and growth conditions

Bacterial strains and plasmids used in this study are listed in Supplementary Table S1. *E. meliloti* and *R. leguminosarum* bv. trifolii strains were routinely grown in TY^30^ or YMA media^31^ at 28 °C. Antibiotics were added as required at the following concentrations: kanamycin 50 µg/ml (*Rhizobium* strains) or 200 µg/ml (*Ensifer* strains) and tetracycline at 10 µg/ml.

### Plant assays and growth conditions

White clover (*T. repens* L. var HUIA) and alfalfa (*M. sativa* L. var. Aragon) seeds were surface-disinfected with 70% ethanol for 30 s and 5% NaClO for 2 min. Seeds were subsequently washed several times and laid on 1% water-agar plates in the dark until germination. *M. truncatula* Gaertn. Jemalong A17 and *nfp-1* mutant^32^ seeds were scarified, surface-disinfected and germinated as described^33^.

For infection assays, 2-day-old seedlings with similar root lengths were selected and transferred to square plates (10 × 10 cm), containing nitrogen-free Fähraeus agar medium^34^ overlaid with a sterile filter paper. Each seedling was inoculated with 200 µl of an OD_600nm_ equivalent to 0.5 of bacterial stationary cultures. Plates were incubated vertically in a growth chamber at 20–24 °C and a 16:8 h photoperiod.

### Root hair microscopy

20-day-old *Trifolium* and *Medicago* seedlings were microscopically visualized under isotonic conditions. Root hairs were examined by brightfield microscopy (Leica FW400 microscope) and phenotypes occurring in primary infection per cm of root were quantified. A Leica TCS SP2 confocal scanning microscope was used to monitor the derivative strains tagged with green fluorescence protein (GFP) using a blue excitation filter (excitation maximum 488 nm; 530-nm long-pass filter).

### ß-galactosidase and NBT (nitroblue tetrazolium) staining

To monitor root hair pre-infection and infection events, *M. truncatula* A17 and *nfp-1* mutant roots were collected 6 days post-inoculation with *lacZ*-tagged bacteria for ß-galactosidase staining as previously described^35,36^. Briefly, entire roots were fixed with 2.5% (V/V) glutaraldehyde (Merck) in Z’ buffer [sodium phosphate buffer (1 M NaH_2_PO_4_ + Na_2_HPO_4_, pH7), 1 M KCl and 1 M MgSO_4_] for 1 h and then rinsed three times in Z’ buffer. Roots were transferred to staining solution [Z’ buffer containing 2% X-gal (5- bromo-4-chloro-3-indolyl-β-D-galactoside), 5 mM K_3_Fe(CN)_6_, and 5 mM K_4_Fe(CN)_6_] and incubated overnight at 28 °C in the dark.

ROS, particularly superoxide (O_2_^−^), accumulation in the root hair tips was observed by nitroblue tetrazolium (NBT) staining as previously described^37^. Roots were immersed in 0.1% NBT (Sigma) for 30 min and subsequently observed by brightfield microscopy, using a Leica FW400 microscope as described above.

### Root hair isolation and gene expression analyses

Root hairs cells were harvested as described in Breakspear *et al*.^38^. RNA isolation from collected root hairs was performed with the RNeasy micro kit (Qiagen), according to the manufacturers protocol. Residual DNA was removed with DNAse I (Thermo). First strand cDNA synthesis was carried out using the High Retrotranscriptase Kit (Biotools) with 1 µg of DNA-free root hair RNA.

End-point RT-PCR reaction was performed in MyCycler (BioRad) on 30-fold diluted cDNA using the REDExtract-N-Amp PCR Reaction Mix (Sigma). The same cDNA was used for qPCR using the Power SYBR Green PCR Master Mix (Applied Biosystems, Life Technologies), following the manufacturer’s recommendations in ABI PRISM 7000 (Applied Biosystems, Life Technologies). Amplification of a *rip1* (Medtr5g074860) gene 160 bp fragment was carried out using primers RIP1F: 5′-GATGCAAGAACAGCAAGCAA-3′ and RIP1R: 5′-AGTGTGGCCACCAGAAAGAG-3′. All results were standardized to the Histone-3-like (Medtr4g097170) expression levels (primers EF1αF; 5′-CTTTGCTTGGTGCTGTTTAGATGG-3′ and EF1αR; 5′-ATTCCAAAGGCGGCTGCATA-3′) as previously described^39^. The 2-∆∆C^t^ method^40^ was applied to determine relative gene expression.

## Results

### Heterologous expression of CelC2 cellulase in ***E. meliloti*** elicits Hole on the Tip (HoT) phenotype in ***T. repens***

**Figure 1:**
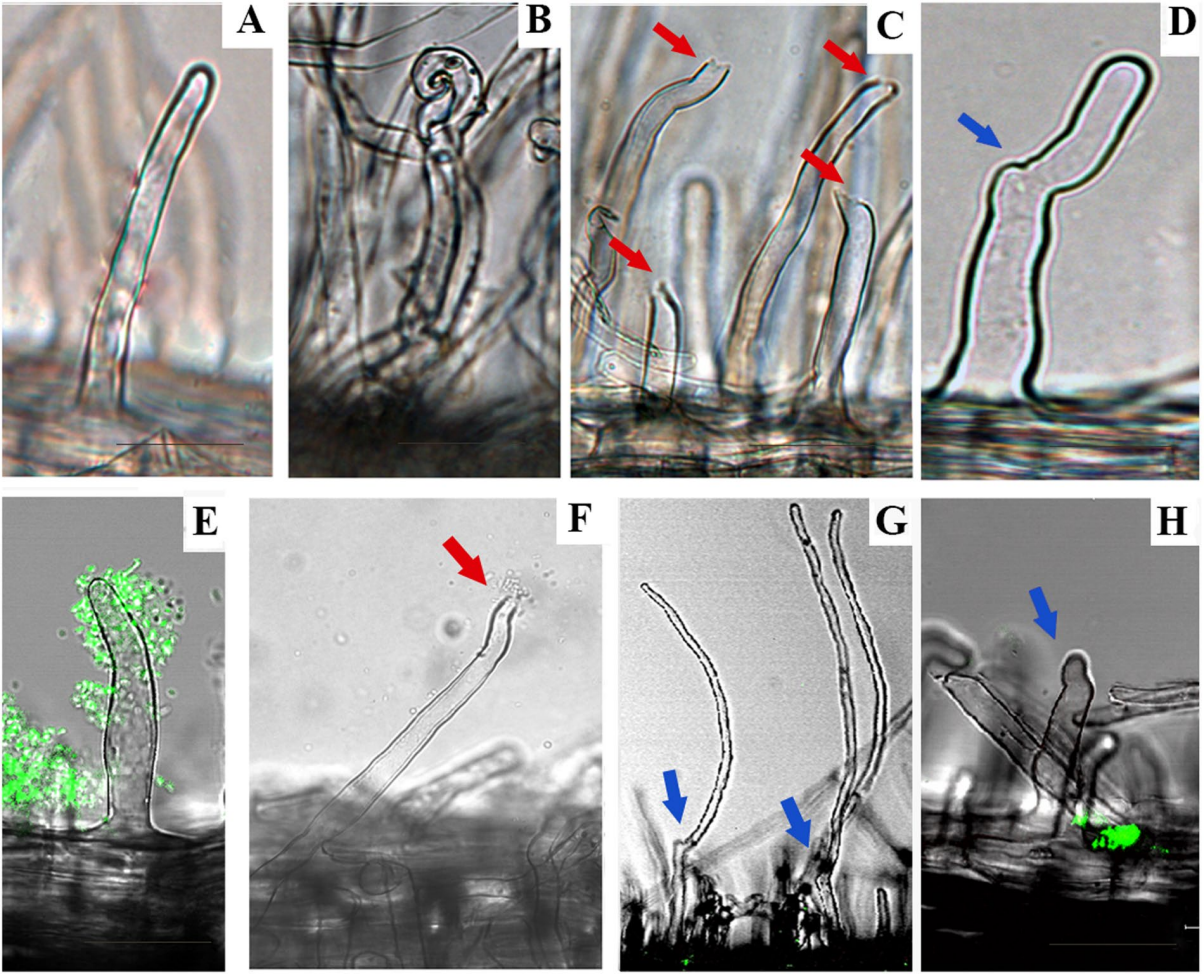
Symbiotic phenotypes in primary infection of *T. repens* root hairs. Brightfield (**A–D,F,G**) and confocal epifluorescence (**E,H**) micrographs showing primary infection events. Plants were inoculated with *R. leguminosarum* bv trifolii ANU843 (**B**), ANU843C2^+^ (**C,D**), *E. meliloti* 1021 (**E**) and 1021C2^+^ (**F–H**). Panel A. Root hairs of uninoculated plants. Bars 20 µm (**A–G**) and 40 µm (**H**). Red and blue arrows indicate HoT (Hole on the Tip) and RaT (Redirections at the Tip) symbiotic phenotypes examples, respectively.

Previous studies showed that the purified cellulase CelC2 isozyme from wild-type ANU843 degrades the cell wall at the apex of the root hair tip (“HoT” phenotype) when incubated with intact seedling roots of its compatible host, white clover^10^. *E. meliloti* 1021 is capable of infecting and nodulating *Medicago* but not *Trifolium* and its genome does not encode CelC homologs^16^. To assess CelC2 effects on primary infection of a non-cognate legume host, *T. repens* seedlings were inoculated with *E. meliloti* wild-type or *celC*-overexpressing (1021C2^+^) strains. Similar symbiotic tests were performed with the clover-compatible ANU843 strain or its overexpressing CelC2 derivative (CelC2^+^). Canonical symbiotic infection phenotypes occurring at early stages of rhizobia-root hair recognition, namely Hac (Hair curling) and noi (nodule initiation), together with Hole on the Tip (HoT) were monitored and documented with a digital microscope (Fig. 1, Table 1).

**Table 1:** Symbiotic phenotypes displayed by *T. repens* roots hairs after inoculation with *R. leguminosarum* bv trifolii ANU843, *E. meliloti* 1021 and their CelC2 derivatives at 20 days post-inoculation.

	Hac/cm*	HoT/cm	RaT/cm	Root length (cm)^**^	noi per root*
Uninoculated	0.0 ± 0.00^a^	1.7 ± 0.28^a^	1.4 ± 0.57^a^	2.2 ± 1.76	0.0 ± 0.00
*R. leguminosarum* bv trifolii ANU843	3.1 ± 0.69^b^	0.2 ± 0.08^a*^	1.6 ± 0.33^a^	2.1 ± 0.85	16.0 ± 2.50
*R. leguminosarum* bv trifolii ANU843C2^+^	2.1 ± 0.29^b^	3.8 ± 0.66^b*^	13.8 ± 3.43^b^	1.6 ± 0.75	13.0 ± 2.61
*E. meliloti* 1021	0.0 ± 0.00	4.2 ± 0.22^a^	3.4 ± 0.51^a^	1.8 ± 1.03	0.0 ± 0.00
*E. meliloti* 1021C2^+^	0.0 ± 0.00	9.3 ± 1.28^b^	9.49 ± 1.78^b^	2.2 ± 0.95	0.0 ± 0.00

Values are the mean ± SE of at least 6 repetitions per treatment. Values followed by different letters (a, b) are significantly different from each other (considering each column and strain-host set) at P < 0.05, according to Fisher’s Protected LSD (least-significant differences) statistic test. Hac (Hair curling), HoT (Hole on the Tip), and RaT (Redirections at the Tip) events per cm of root. noi (nodule initiation).

^*^Data from Robledo *et al*. 2011^12^.

^**^Values represent the mean ± SD.

Uninoculated *T. repens* roots (Fig. 1A) presented intact root hairs with no sign of rhizobial infection events whereas those inoculated with the ANU843 wild type bacteria (Fig. 1B) presented typical compatible infection events like root hair curling and IT formation. Matching previous results^12^, clover roots inoculated with ANU843C2^+^ derivative showed an increased number of HoT events (Fig. 1C, red arrows), indicating an extensive degradation of the noncrystalline cellulose located at root hair tips. Moreover, we also observed in these samples a root hair-associated phenotype characterized by redirections of root hair growth occuring at the root hair apex (Fig. 1D, blue arrow). These branches in the root hairs seem to be formed by deposition of new cell wall material following hydrolysis of the initial root hair tip, leading to redirection of a new tip, which is displaced from the original cell axis^12^. This phenotype will be referred to as RaT (Redirections at the Tip). ANU843C2^+^-inoculated clover roots showed an 8.6-fold increase in the number of RaT events per cm compared with those inoculated with ANU843wt strain (Table 1).

On the other hand, even though *E. meliloti* 1021 actively colonized root hair tips, it was unable to induce ITs or nodule primordia in its non-host *T. repens* (Fig. 1E). An *E. meliloti* 1021 derivative transformed with EV (empty-vector) was also included as control in the infection assays, showing no evident differences compared with the wild-type strain (data not shown). Interestingly, 1021C2^+^ promoted a significant increase of both HoT (2.2-fold; Fig. 1F, red arrows) and RaT (2.7-fold; Fig. 1G,H, blue arrows) events with respect to those recorded using 1021 (Table 1). Therefore, expression of *celC* gene likely conferred *E. meliloti* the ability to elicit early host-specific symbiotic responses underlying degradation of the noncrystalline cellulose at root hair tips for primary plant infection.

### *Medicago* plants display a significant increase in root hair tip redirections (RaTs) upon inoculation with CelC2-overproducing strains

**Figure 2:**
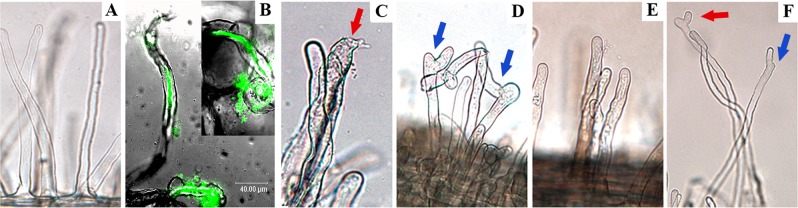
Symbiotic phenotypes in primary infection of *M. sativa* root hairs. Brightfield (**A,D–F**) and confocal epifluorescence (**B**) micrographs showing primary infection events on *M. sativa* root hairs. Uninoculated root hairs are shown in panel A. Plants were inoculated with *E. meliloti* 1021 (B, C), 1021C2^+^ (**D**), *R. leguminosarum* bv. trifolii ANU843 (**E**) and ANU843C2^+^ (**F**). Bars 20 µm (**A,C–F**) and 40 µm (**B**). Red arrows indicate HoT symbiotic phenotypes. Blue arrows indicate RaT symbiotic phenotypes.

Reciprocally, we inoculated *M. sativa* seedlings with ANU843, 1021 or the corresponding *celC*^+^ derivative strains. Unlike in mock-treated roots (Fig. 2A), *E. meliloti* 1021 induced in alfalfa the typical root hair deformation (Fig. 2B) and nodule initiation (noi) phenotypes displayed by a compatible rhizobia-legume symbiosis (Table 2). *M. sativa* inoculated with 1021 CelC2-producing derivative strain showed a similar number of Hac and HoT events in comparison to wild-type inoculated roots (Table 2; Fig. 2B,C). However, a significant higher number of RaT (around 3.5-fold) events was recorded in seedlings inoculated with 1021C2^+^ strains compared to the uninoculated and 1021-inoculated ones (Fig. 2D, blue arrows).

**Table 2:** Symbiotic phenotypes of *M. sativa* root hairs after inoculation with *E. meliloti* 1021, *R. leguminosarum* bv trifolii ANU843 and their CelC2 derivatives.

	Hac/cm	HoT/cm	RaT/cm	Root length (cm)	noi per root
Uninoculated	0.0 ± 0.00^a^	0.3 ± 0.04ª	1.4 ± 0.28ª	6.7 ± 1.85	0.0 ± 0.00
*E. meliloti* 1021	1.9 ± 0.16^b^	1.9 ± 0.48^a^	1.37 ± 0.55ª	4.7 ± 1.80	3.0 ± 1.22
*E. meliloti* 1021C2^+^	1.9 ± 0.08^b^	1.4 ± 0.14^a^	5.3 ± 0.61^b^	4.25 ± 1.93	2.0 ± 1.00
*R. leguminosarum* bv trifolii ANU843	0.0 ± 0.00a	2.6 ± 0.44^b^	4.5 ± 0.53ª	4,5 ± 1,50	0.0 ± 0.00
*R. leguminosarum* bv trifolii ANU843C2^+^	0.0 ± 0.00^a^	2.1 ± 0.45^b^	9.5 ± 0.43^b^	3.0 ± 0.93	0.0 ± 0.00

Measurements have been performed and analyzed as described in Table 1.

Cross-inoculation of alfalfa roots with *R. leguminosarum* bv. trifolii ANU843 showed no Hac or noi events although bacterial cells attached to root hairs (Fig. 2E). In ANU843C2^+^-alfalfa inoculated roots, RaT phenotype becomes evident (Fig. 2F, blue arrows), showing a significant increase in comparison to ANU843-inoculated (2.2-fold) and uninoculated plants (6.5-fold). Together, these findings identified the increase of root hair branches as a CelC2-dependent early symbiotic host-unspecific response.

### Medicago plants impaired in IT development showed more root hair redirection at the tip (RaT) events than A17 upon 1021C2^+^ inoculation

**Figure 3:**
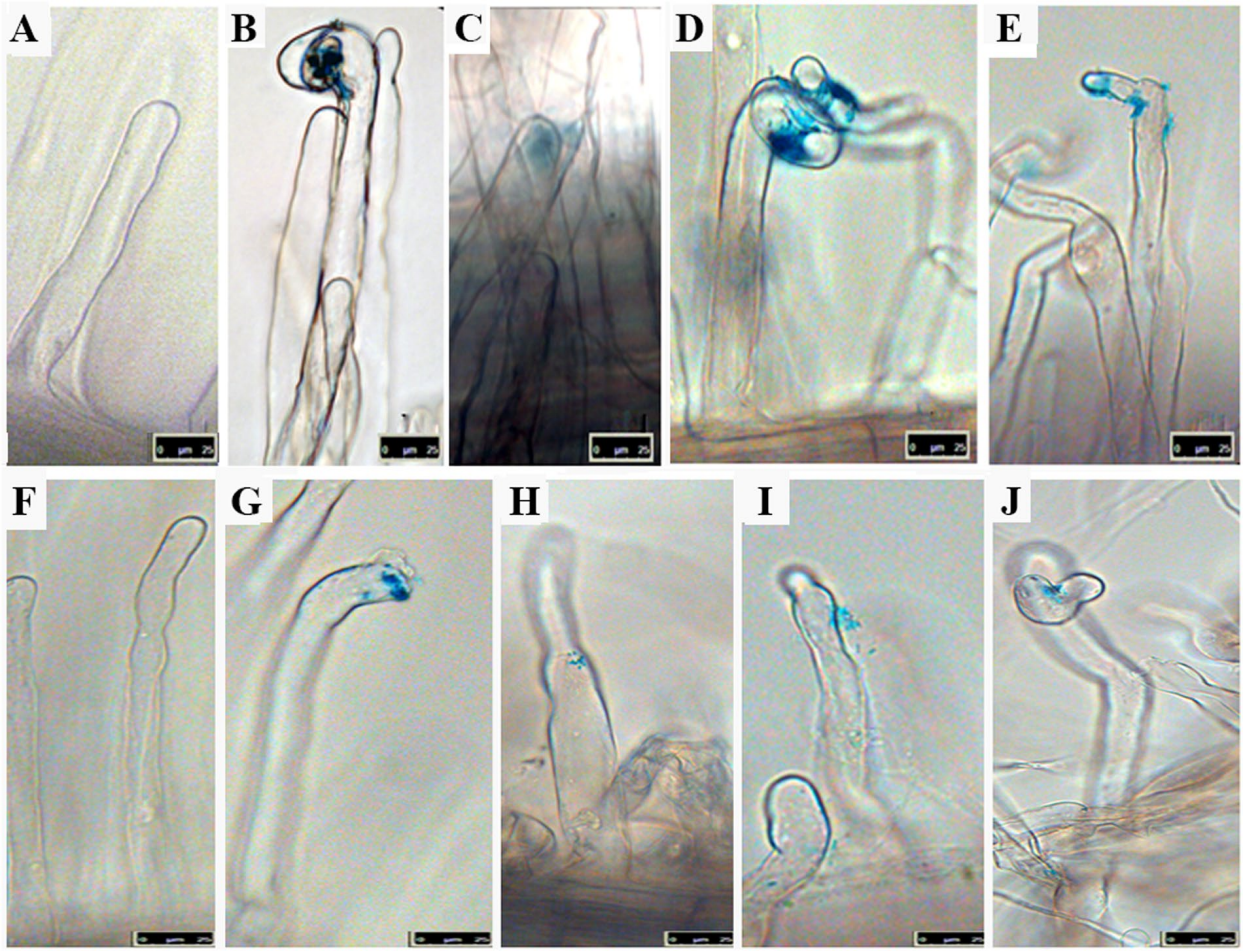
Symbiotic phenotypes in primary infection of *M. truncatula* A17 (**A–E**) and *nfp-1* mutant (**F–J**) root hairs (6dpi). Plant roots were inoculated with *E. meliloti* 1021 (**B,C,G,H**) and 1021C2^+^ (**D,E,I,J**), both harbouring pXLGD4 plasmid (lacZ staining). (**A,F**) Micrographs correspond to uninoculated plants of A17 and *nfp-1* varieties, respectively. Bars 25 µm.

Infection assays were also performed in the model legume *M. truncatula*. This model legume establishes a compatible symbiotic interaction with *E. meliloti* 1021, although not so efficient in terms of nitrogen fixation than with *M. sativa*^41^. *M. truncatula* provides a plethora of resources for the study of its interaction with rhizobia. i.e. the *nfp-1* mutant, which is impaired in infection thread formation due to blocking of the early signalling cascade^32^. *M. truncatula* A17 (Fig. 3A–E) and *nfp-1* mutant derivative plants (Fig. 3F–J) were inoculated with *E. meliloti* 1021 and 1021C2^+^ strains tagged with *lacZ* as reporter. Both *M. truncatula* uninoculated lines showed regular root hair structures (Fig. 3A,F). A17 inoculated with 1021 exhibited typical attachment to the root hair apex and curls (Hac phenotype) symbiotic phenotypes (Fig. 3B,C). Hac events also appeared in A17-1021C2^+^ inoculated roots, but were fewer in number (Fig. 3D, Table 3). In agreement with the *M. sativa* results, there was a significant increase in the number of events classified as RaT (Fig. 3E), which were more frequently found (~5.8-fold) than in 1021-inoculated and mock-treated plants.

**Table 3:** Symbiotic phenotypes in *Medicago truncatula* A17 and *nfp-1* root hairs 6 days after inoculation with *E. meliloti* 1021 and its CelC2-expressing derivative.

	Strain	Hac/cm	HoT/cm	RaT/cm
*M. truncatula* A17	Uninoculated	0.0 ± 0.00^a^	0.3 ± 0.14ª	0.4 ± 0.08^a^
	*E. meliloti* 1021	1.4 ± 0.65ª	0.3 ± 0.05^a^	0.7 ± 0.11^a^
	*E. meliloti* 1021C2^+^	0.3 ± 0.11^a^	1.8 ± 0.75^a^	2.9 ± 0.43^b^
*M. truncatula nfp-1*	Uninoculated	nd	nd	0.2 ± 0.04^a^
	*E. meliloti* 1021	nd	nd	0.5 ± 0.07^a^
	*E. meliloti* 1021C2^+^	nd	nd	5.3 ± 0.24^b^

Values are the mean ± SE of at least 6 repetitions per treatment. Values followed by different letters (a, b) are significantly different at P < 0.05, according to Fisher’s Protected LSD (least-significant differences) statistic test. Hac (Hair curling). HoT (Hole on the Tip). RaT (Redirections at the Tip). nd (no data/determined).

As expected, all *nfp1*-inoculated roots presented microcolonies adhered to the root hairs, with no signs of rhizobial invasion or infection thread formation and no evident Hot phenotype (Fig. 3G–J). In contrast, the number of RaT phenotype events in 1021C2^+^-inoculated *nfp-1* plants (Fig. 3I,J) significantly increased (~10-fold) with with respect to *nfp-1* control, to 1021-inoculated roots and to A17 inoculated with 1021C2^+^ (2-fold; Table 3). This result suggest that the RaT phenotype occurs independently of the plant ability to proceed with later infection stages, such as IT formation.

### Heterologous CelC2 production induces oxidative burst on *Medicago* root hairs at early infection stages

**Figure 4:**
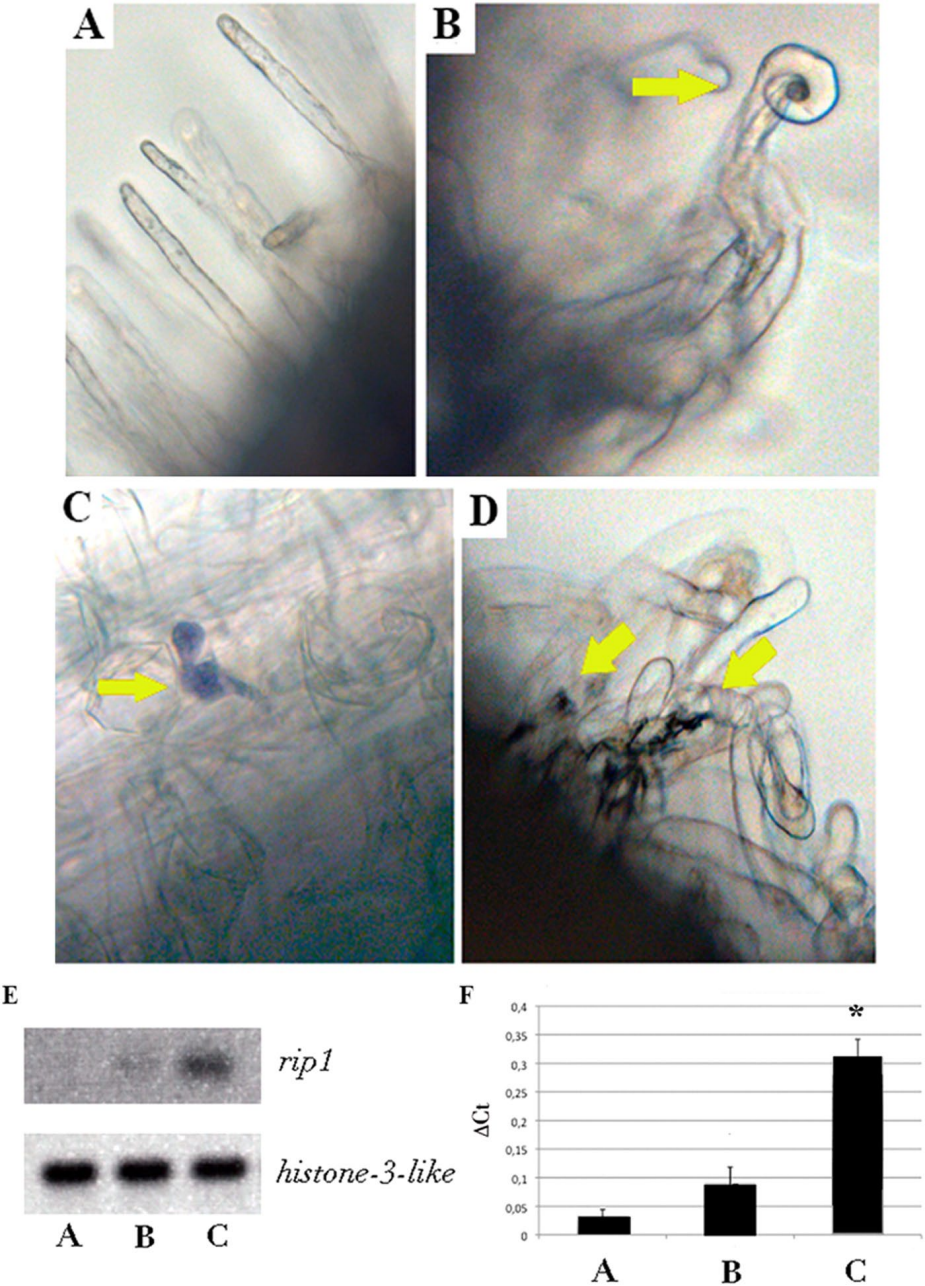
ROS-related responses on *M. truncatula* root hair tips. Brightfield micrographs after NBT staining of *M. truncatula* uninoculated root hairs (**A**) and 6 days post inoculation with *E. meliloti* 1021 (**B**) or 1012C2^+^ (**C**,**D**). Bars 20 µm. Yellow arrows show NBT-stained zones (superoxide). End-point RT-PCR (**E**) and qPCR (F) assays of *M. truncatula rip1* gene expression in root hairs of (**A**) uninoculated plants, **(B**) *E. meliloti* 1021 and (**C**) *E. meliloti* 1021C2^+^. Results were normalized with the histone-3-like (H3L) housekeeping gene. Data presented is the average of biological 3 replicates for each treatment. Bars represent SE. Asterisk indicates statistically different means P < 0.01, according to Fisher’s Protected LSD (least-significant differences) statistic test.

Our previous studies also reported transient ROS accumulation and reduced calcium spiking signalling upon alteration of CelC2 levels in the rhizobial partner^12,13^, suggesting a role of this enzyme in symbiotic specificity. To elucidate whether CelC2 cellulase triggers changes in ROS production during the *E. meliloti-M. truncatula* interaction, we first performed NBT staining assays to detect plant production of a specific subtype of ROS (i.e. superoxide)^42^. Similar to mock-treated roots, 6 days post-inoculation with wild-type 1021 strain, *M. truncatula* A17 showed no signs of intense oxidative reaction at the infection sites (Fig. 4A,B, green arrow). However, there was slight root hair coloration in root cells inoculated with strain 1021C2^+^ at this time point (Fig. 4C,D, green arrows), indicating an increase in superoxide production.

To confirm these results, we isolated RNA from root hairs and performed end-point RT-PCR and qPCR with primers to amplify *Rhizobium Induced Peroxidase 1 (rip1)*, an early nodulin known to be transiently expressed in sites with localized production of ROS^37,43^. The results showed a significant induction of *rip1* expression 6 days after inoculation with 1021C2^+^ with respect to uninoculated or 1021-inoculated control roots (Fig. 4E.F). We therefore conclude that CelC2 production elicits a continuous expression of an early nodulin in non-compatible legume hosts.

## Discussion

The so-called CelC2 cellulase is a symbiotic endoglucanase originally described in *R. leguminosarum* bv. trifolii, a *Rhizobium* species that specifically nodulates white clover. CelC2 has two described functions: (i) it modulates the length of cellulose microfibrils, affecting biofilm formation, and (ii) it catalyses the localized hydrolysis of the root cell-wall, thereby promoting primary and secondary infection into the legume host tissues^9,10,12,16,44,45^. Moreover, a recent study confirmed an impact of this cellulase in earlier stages of symbiotic signalling, interfering with calcium spiking and delaying nodulation^13^. In the present work, we used wild-type and *celC*-overexpressing variants from rhizobial species encoding CelC2 (*R. leguminosarum* bv trifolii ANU843) or lacking *celC* homologs in the genome (*E. meliloti* 1021), to evaluate effects of CelC2 on primary infection of their compatible and non-cognate legume hosts. Moreover, we took advantage of the genetic tools provided by *M. truncatula* and *E. meliloti* to further investigate the CelC2 activity at the molecular level in the first stages of the interaction.

Despite CelC2’s role in degradation of the material located at the root hair tip to provide the portal of entry of rhizobia in the *R. leguminosarum-T. repens* symbiosis, there were some unanswered questions regarding additional (downstream) molecular responses of host root hair cells to CelC2 activity. What was already known, and supported by our present results, is that purified CelC2 enzyme cannot induce HoT phenotype or even erode root hair tips of *M. sativa*^9,10^. The increase in the number of HoT events, when CelC2-producing strains are inoculated on white clover, confirmed that this cellulase has a strong specificity for substrates located at the root hair apex, suggesting that this specificity is related with the root hair tip cell-wall composition and/or architecture. Composition of the root hair cell-wall seems to differ from that of other root cells^46^ in terms of cellulose microfibrils distribution, texture and deposition during root hair growth^47,48^ and also, in terms of crystallinity changes in the isotropic architecture at the apex cell-wall^49^. Our findings support the already described role of a well-balanced production of rhizobial cellulases for the localized penetration of compatible rhizobia into root hairs of specific legume hosts.

Even though control or wild-type inoculated plants showed some HoT events, possibly resulting from mechanical damage, only a few of the tip-hydrolysed root hairs are able to continue their growth forming polar and sub-polar redirections, while other roots showed a larger proportion of degraded root hair tips. Interestingly, we also observed a new phenotype based on redirections of growth occurring at the root hair tip. This phenotype is significantly found more frequently when roots are inoculated with CelC2 overexpressing rhizobia, regardless of the plant-bacterial combination used. These results suggest that non-host legumes may be susceptible to root hair hydrolysis mediated by rhizobial endoglucanases to different extents and that such a feature is not restricted to clover.

Cellulase activity acidifies and depolarizes membranes^50^, effects that can be responsible for the observed redirections of root hair tip growth. When bacteria are bound to the root hair tip, the effects of cellulase CelC2 seem to be more localized than upon addition of the purified enzyme, in agreement with the fact that it is cell-bound^51^. Our results suggest that these non-specific membrane depolarisations and zone acidification could be attributed to the action of higher levels of CelC2 cellulase.

Data obtained with the *M. truncatula*- *E. meliloti* symbiotic system confirmed the results obtained in *T. repens* and *M. sativa* inoculated with their compatible strains, supporting the hypothesis that the RaT phenotype is a common event driven by the expression of rhizobial endoglucanases. Additionally, the assays performed with *M. truncatula nfp-1* mutants, impaired in infection and nodulation^30^, also showed an increase of RaT events upon inoculation with CelC2 overproducing derivatives, suggesting that the action of CelC2 cellulase is independent of the host ability to perceive NFs. It is also worth noting that this cellulase does not degrade the *E. meliloti* NF backbone^13^. These findings support that RaT phenotype is different to other phenotypes observed after wild-type rhizobial inoculation and/or NF root hair treatments, such as root hair branching and root hair tip swelling^32,52^.

ROS/NO homeostasis has been reported to modulate bacterial-legume specificity. Changes on the production of these signals can hamper early steps of symbiosis development. While ROS levels mainly impact root hair curling and ITs formation, NO exerts control over nodule development^7^. Some authors reported that there is a transient oxidative burst upon rhizobial infection, which results in ROS accumulation at the infection sites^13,38,53^. Our previous work^13^ reported transient changes of ROS accumulation on *T. repens* root hair tips upon infection with strain ANU843C2^+^. Moreover, Lohar *et al*.^52^ described that root hair branching was also related to transient changes of ROS levels.

In our present analyses, NBT (nitroblue tetrazolium) staining revealed that there is a certain induction of superoxide production in *M. truncatula* root hairs inoculated with the 1021C2^+^ strain. Our results showed there are slight effects, but still they are comparable with previously published results on *T. repens* root hairs response to CelC2 overexpression^12^. These authors used a ROS-sensitive fluorescent dye (H_2_DCFDA), which is a general cellular oxidative redox marker rather than a detector of specific types of ROS^54^, in contrast with the superoxide-sensitive NBT staining used in our case of study. Nevertheless, it is also conceivable that ROS accumulation could be continuous upon CelC2 overexpression but also not enough to totally block infection. This misregulation of ROS homeostasis produced by the extra amount of CelC2 seems to be enough to induce the redirection of the polar growth of the root hair tips but does not totally block infection.

Peroxidases catalyse the formation of ROS, which are related to cell wall loosening in growing cells^55^. To confirm the elicitation of ROS production by CelC2, we tracked expression of the *RIP1* gene, a peroxidase that was originally described as induced by the presence of *Rhizobium*^56^. This gene is induced in *M. truncatula* within the first hours upon *E. meliloti* infection, and is downregulated 48 hours after inoculation^38,56–59^. This expression profile correlates with ROS production elicited by NFs^43,60^.

The *M. truncatula* “infectome” database revealed that *Rhizobium-Induced Peroxidase* genes (*RIP1-10*) are induced in root hairs upon rhizobial infection or NF addition^38,61^. Moreover, recent reports showed that some of these *RIP* genes are also induced in different zones of the nodule, mostly at the nodule apex where cells are growing and expanding^61,62^. Our results indicated that *rip1* is expressed at low levels on 1021-inoculated *M. truncatula* root hairs at 6 days post-inoculation in comparison with non-inoculated root hairs. The presence of *RIP1* gene expression at 6 days post-inoculation with the *celC*^+^ derivative and the detection of ROS levels may indicate that the effect of cell wall loosening is maintained at these points (growing apex). This means that the noncrystalline cellulose (typically found in growing cells, such as the root hair apex) is continuously available for CelC2 action, which is likely connected to the delay in nodulation experienced by plants inoculated with *celC*^+^ derivatives in *Rhizobium–Trifolium* and *Ensifer-Medicago* symbiotic systems^12,13^. The continuous state of cell wall loosening induced by extra levels of CelC2 expression is likely the cause of the increased number of RaT events and might explain the alteration of the signalling cascade, also supported by the results showed by Robledo *et al*.^13^, where cellulase CelC2 does not cleave NFs.

In conclusion, our results confirm that the *R. leguminosarum* bv trifolii ANU843 CelC2 cellulase has a strong substrate specificity for the noncrystalline cellulose located at the root hair tips of its specific host *T. repens*. The substrate specificity is the key to the success of the canonical primary infection in *Rhizobium-Trifolium* symbiosis. Interestingly, CelC2 cellulase promotes the appearance of growth redirections in the root hairs: the newly-named RaT phenotype, which is common to cognate and non-cognate hosts. Moreover, our data demonstrate that extra levels of CelC2 cellulase lead to alterations in the primary infection points, confirming the importance of this cellulase in the symbiotic process. Further studies are needed to decipher the mechanisms underlying specific and non-specific early symbiotic responses of the legume hosts to rhizobial cellulases.

## Supplementary information

Supplementary Information

## Data Availability

The authors declare that all the data supporting the findings of this study are available within the article and its Supplementary Information File.
