# Supplementary material for: Legumes display common and host-specific responses to the rhizobial cellulase CelC2 during primary symbiotic infection
Source: Sci Rep. 2019 Sep 25;9:13907. doi: 10.1038/s41598-019-50337-3 (PMC6761101; doi:10.1038/s41598-019-50337-3)

SUPPLEMENTARY INFORMATION

Title: Legumes display common and host-specific responses to the rhizobial cellulase CelC2 during primary symbiotic infection

Authors: Menéndez E.[1, 2¥*], Robledo M.[1, 3Ŧ*], Jiménez-Zurdo JI[3], Velázquez E[1, 4], Rivas R[1,2,4], Murray JD[5Ť] and Mateos PF[1,2,4#].

[1] Departamento de Microbiología y Genética, Universidad de Salamanca, Spain.

[2] Instituto Hispano-Luso de Investigaciones Agrarias (CIALE), Universidad de Salamanca, Spain.

[3] Estación Experimental del Zaidín, CSIC, Granada, Spain

[4] Unidad asociada de I+D IRNASA-CSIC, Salamanca, Spain.

[5] Department of Cell and Development Biology, John Innes Centre, Norwich, UK

[#] Corresponding author: Dr. Pedro F Mateos

Department of Microbiology and Genetics/CIALE

Ed. Departamental Lab. 213 Campus de Unamuno

University of Salamanca

C/ Doctores de la Reina s/n

37007 Salamanca, Spain

Phone: +34923294500 ext 1911

pfmg@usal.es

*These authors contributed equally to this work

¥ Current affiliation: Instituto de Ciências Agrárias e Ambientais Mediterrânicas (ICAAM), Universidade de Évora, Évora, Portugal.

Ŧ Current affiliation: Instituto de Biomedicina y Biotecnología de Cantabria, Santander, Spain.

Ť Current affiliation: Centre of Excellence for Plant and Microbial Science, Shangai, China.

Table S1.

Bacterial strains and plasmids used in this study.

Strains	Characteristics	Reference
Ensifer meliloti		
1021	*E. meliloti* SU47 Str^r derivative	63
1021EV	Control derivative of 1021 containing the pBBR1MCS-2 plasmid (empty vector), Km^r	13
1021C2$^+$	1021 containing pJZC2 plasmid expressing celC gene; Km^r	13
1021GFP	1021 containing pHC60 plasmid; Tc^r	This work
1021C2$^+$GFP	1021 containing pJZC2 and pHC60 plasmids; Km^r, Tc^r	This work
1021lacZ	1021 containing pXLGD4 plasmid; Tc^r	36
1021C2$^+$lacZ	1021 containing pJZC2 and pXLGD4 plasmids; Km^r, Tc^r	This work
Rhizobium leguminosarum bv trifolii		
ANU843	Wild-type Fix+ Nod+	64
ANU843EV	Control derivative of ANU843 containing the pBBR1MCS-2 plasmid (empty vector) Km^r	12
ANU843C2$^+$	ANU843 containing pJZC2 plasmid expressing celC gene; Km^r	12
ANU843GFP	ANU843 containing pHC60 plasmid; Tc^r	12
ANU843C2$^+$GFP	ANU843 containing pJZC2 and pHC60 plasmids; Km^r, Tc^r	12
Escherichia coli		
DH5α pXLGD4	Reporter plasmid carrying the hemA::lacZ fusion, Tc^r	65
S17.1 pHC60	thi pro hsdR– hsdM+ recA RP4 2-Tc::Mu-Km::Tn7 (Sp^r /Sm^r)<br><br>Contains gfp-expressing derivative of pHC41 plasmid; Tc^r	66

References

12. Robledo, M. *et al.* Development of functional symbiotic white clover root hairs and nodules requires tightly regulated production of rhizobial cellulase CelC2. *MPMI* **24,** 798–807 (2011).

13. Robledo, M. *et al.* Heterologous Expression of Rhizobial CelC2 Cellulase Impairs Symbiotic Signaling and Nodulation in Medicago truncatula. *MPMI* **31,** 568–575 (2018).

36. Boivin, C., Camut, S., Malpica, C. A., Truchet, G. & Rosenberg, C. *Rhizobium meliloti* Genes Encoding Catabolism of Trigonelline Are Induced under Symbiotic Conditions. *Plant Cell* **2,** 1157–1170 (1990).

63. Meade, H. M., Long, S. R., Ruvkun, G. B., Brown, S. E. & Ausubel, F. M. Physical and genetic characterization of symbiotic and auxotrophic mutants of *Rhizobium meliloti* induced by transposon Tn5 mutagenesis. *J. Bacteriol.* **149,** 114–122 (1982).

64. Rolfe, B. G., Gresshoff, P. M., Shine, J. & Vincent, J. M. Interaction Between a Non-Nodulating and an Ineffective Mutant of *Rhizobium trifolli* Resulting in Effective (Nitrogen-Fixing) Nodulation. *Appl. Environ. Microbiol.* **39,** 449–452 (1980).

65. Cheng, H. P. & Walker, G. C. Succinoglycan is required for initiation and elongation of infection threads during nodulation of alfalfa by *Rhizobium meliloti*. *J. Bacteriol* **180,** 5183–5191 (1998).

66. Leong, S. A., Williams, P. H. & Ditta, G. S. Analysis of the 5' regulatory region of the gene for delta-aminolevulinic acid synthetase of Rhizobium meliloti. Nucleic acids research, **13**, 5965–5976. (1985).

Supplementary Figure S1

Full-length gel used to generate Fig. 4E

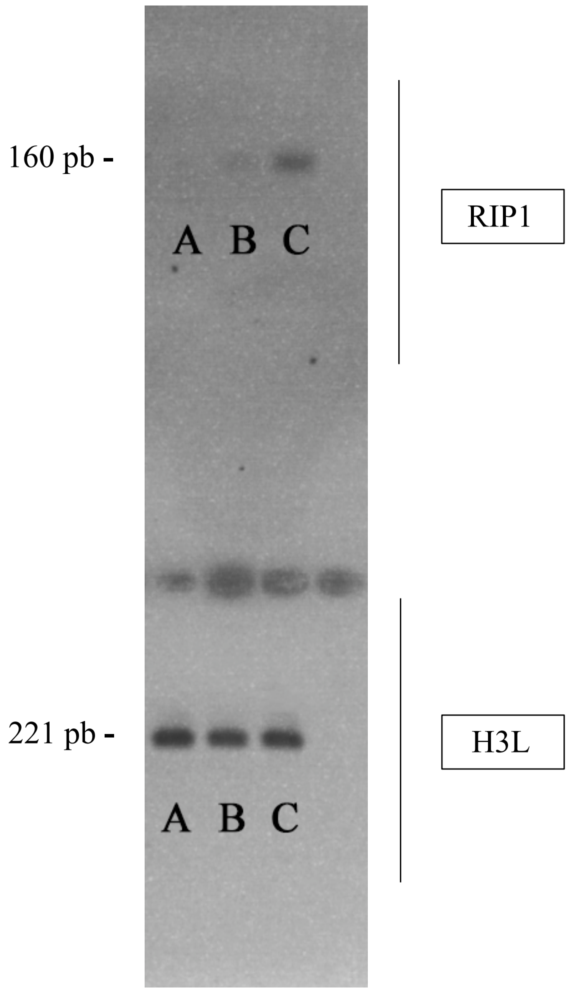

Supplement: Supplementary file 1 — Supplementary Information [file 41598_2019_50337_MOESM1_ESM.pdf]
